# Human Papillomavirus vaccine knowledge and recommendation practice among primary care providers, Almaty, Kazakhstan – 2023

**DOI:** 10.1080/21645515.2025.2610622

**Published:** 2026-02-02

**Authors:** Feruza Ablimitova, Dilyara Nabirova, Saya Gazezova, Manar Smagul, Ainagul Kuatbaeva, Aizhan Yesmagambetova, Alexander J. Millman, Roberta Horth

**Affiliations:** a Central Asia Field Epidemiology Training Program, Department of Medical and Preventive Care, Asfendiarov Kazakh National Medical University, Almaty, Kazakhstan; b Scientific and Practical Center for Sanitary and Epidemiological Expertise and Monitoring, Almaty, Kazakhstan; c Division of Global Health Protection in Central Asia, United States Centers for Disease Control and Prevention, Almaty, Kazakhstan; d Management Board, National Center of Public Health of the Ministry of Health of the Republic of Kazakhstan, Astana, Kazakhstan

**Keywords:** HPV vaccination, papillomavirus vaccine, healthcare workers, HPV infection, cross-sectional study, cervical cancer, Kazakhstan

## Abstract

Kazakhstan planned to reintroduce the human papillomavirus (HPV) vaccine into the national vaccination calendar in 2024 for girls aged 12–14. A 2013 pilot attempt failed due to low acceptance. To inform implementation, we evaluated HPV vaccine knowledge and recommendation practices among primary healthcare workers (HCWs). In April-May 2023, we conducted a cross-sectional survey using convenience sampling among HCWs responsible for vaccination at 5 private and 29 of the largest public polyclinics in Almaty. Participants self-completed anonymous questionnaires. Knowledge scores >70% were considered adequate. We used logistic regression to assess factors associated with intention to recommend HPV vaccines, reporting adjusted odds ratios (OR) and 95% confidence intervals (CI). Among 832 participants, 68% were nurses, and 18% had >20 y experience. One-third (33%) had adequate HPV knowledge, 22% knew HPV has no cure, and 71% understood it is not airborne. One-fifth (20%) could dispel common HPV vaccine myths, 39% dispelled common childhood immunization myths, 61% correctly identified childhood vaccine contraindications, and 58% believed in childhood vaccines’ safety and effectiveness. Overall, 28% would recommend the HPV vaccine to patients or their friends’ children. Doctors were more likely to recommend than nurses (OR = 1.52, 95% CI = 1.06–2.18). Higher recommendation odds were also associated with ability to dispel childhood vaccines myths (OR = 1.47, 95% CI = 1.03–2.07), adequate HPV knowledge (OR = 1.63, 95% CI = 1.14–2.32), belief in vaccine safety (OR = 1.65, 95% CI = 1.12–2.47), and support for vaccinating HCWs (OR = 3.07, 95% CI = 2.11–4.54). HPV-related knowledge and recommendation intent among HCWs were low. Targeted training and communication may improve HPV vaccine uptake in Kazakhstan.

## Introduction

Cervical cancer is the fourth most common cancer in women worldwide, with over 661,000 cases and 348,000 fatalities reported in 2022.^1–3^ In Kazakhstan, approximately 1800 new cases of cervical cancer are reported annually, leading to around 600 fatalities.^4^ Cervical cancer is the second most common cancer among women in Kazakhstan, following breast cancer, and ranks fifth in incidence among all cancer types^1^

The human papillomavirus (HPV) vaccine is a highly effective primary prevention strategy against cervical cancer. Currently licensed vaccines include the bivalent (2vHPV, Cervarix), quadrivalent (4vHPV, Gardasil) and nonavalent (9vHPV, Gardasil-9) HPV vaccines. These vaccines have been linked to a significant reduction in HPV-related diseases, particularly HPV-related cancers, and a decrease in the prevalence of HPV genotypes, all without serious side effects.^5–7^ Furthermore, HPV vaccination programs, combined with cancer screening initiatives, are associated with lower incidence rates and mortality from cervical cancer.^8–12^

Kazakhstan was the first country in Central Asia to introduce the HPV vaccination. In October 2012, the Ministry of Health of Kazakhstan issued an order to include the HPV vaccine in the national immunization schedule for girls aged 12–14 y.^13^ In 2013, Kazakhstan launched a pilot vaccination campaign aimed at immunizing all eligible girls in schools across four regions: Astana city (the capital), Almaty (the most populous city), and two oblasts (Pavlodar and Atyrau). However, the uptake of the vaccines used in this pilot – 4vHPV (Gardasil) and 2vHPV (Cervarix) – was lower than expected. From 2013 to 2016, only 7172 girls received a single dose, and just 4217 completed the full three-dose regimen.^14^ Misinformation and negative perceptions among medical professionals, public figures, and the general public about the vaccine including concerns about vaccine safety and alleged risks of infertility and autoimmune disorders, and effects on sexual behaviors contributed to the low vaccine uptake and the discontinuation of the pilot in 2016^15^ These narratives were amplified through traditional and social media channels in the absence of evidence-based counter-messaging from trusted health authorities. This dynamic may have further undermined public confidence in the vaccine^15^

Low uptake of HPV vaccines is a challenge not only in Kazakhstan but also worldwide. By 2022, 140 (72%) of 194 countries had introduced HPV vaccination programs; however, global coverage for dose completion among eligible girls was 15%.^16^ These figures fall significantly short of the WHO’s Cervical Cancer Elimination strategy, which aims for 90% coverage among adolescent girls by 2030.^17^ Globally, various barriers hinder HPV vaccination, including lack of awareness, safety concerns, cultural beliefs, and hesitancy among healthcare providers.^18^ Children of caregivers who receive accurate information about HPV and HPV vaccines from trusted sources, including their healthcare providers, are more likely to be vaccinated.^19–21^ Additionally, healthcare providers with knowledge and confidence in the vaccines are more likely to recommend them to their patients.^22–24^ However, knowledge gaps among providers regarding HPV transmission, vaccine safety, and eligibility criteria limit their ability to provide recommendations to their patients.^19–21^

Research on HPV vaccine knowledge and uptake in Kazakhstan is limited. A 2022 study conducted among mothers revealed that nearly half (45%) had declined HPV vaccination for their child. Additionally, 50% expressed discomfort discussing the vaccine with their family physicians, and only 3% reported receiving information about the HPV vaccine from their pediatricians.^25^ Despite the central role that primary care workers in Kazakhstan play in promoting vaccination within their communities, there is a notable absence of published studies examining the knowledge, and practices regarding HPV vaccines among Kazakhstani healthcare providers.

In 2024, Kazakhstan reintroduced the HPV vaccine into the national immunization program. A vaccination campaign using the 4vHPV (Gardasil) vaccine was initiated for 11–13-year-old girls, following a two-dose schedule with a six-month interval. The target population included 341,408 girls.^26^ Vaccination was delivered through primary healthcare facilities, with active outreach in schools and community-based settings. Written informed consent from parents or legal guardians was required, and standardized informational materials were provided to families and adolescent girls prior to vaccination. The success of this rollout depended heavily on healthcare providers’ ability to inform their patients and communities and promote vaccine uptake. To support this reintroduction, the present study assessed healthcare workers’ knowledge and practices regarding HPV vaccination.^26^

This study aimed to assess the knowledge and intended practices related to HPV vaccination and childhood immunizations and factors associated with HPV vaccination recommendation behavior among healthcare workers in Almaty, Kazakhstan. The findings were intended to guide the development of educational interventions, national communication strategies, and public health campaigns in preparation for the national vaccine rollout.

## Methods

### Study design and setting

In April and May 2023, we conducted a cross-sectional study across five private polyclinics and the 29 largest (out of 36) public polyclinics providing routine childhood vaccinations in Almaty, a city with a population exceeding two million. As the most populous city in Kazakhstan, Almaty has the largest healthcare infrastructure in the country and participated in the 2013–2016 HPV vaccination campaign pilot. The study was designed and led by residents of the Central Asia Advanced Field Epidemiology Training Program (CA-FETP).

### Study participants

With the support of the Almaty State Department of Health, we identified all general practitioners, pediatricians, and nurses responsible for administering vaccines at the study sites. We aimed to enroll a convenience sample of all eligible vaccination providers at the selected polyclinics. Eligible providers were general practitioners, pediatricians, or nurses aged ≥18 y who were responsible for routine childhood or adolescent vaccinations and present at their workplaces during the survey period. Selected participants who provided written informed consent were interviewed in April and May 2023. Staff not involved in vaccination activities and those who would decline to participate were not part of the survey. No personally identifiable data about participants or their places of work was collected during the interviews.

Of the 1057 eligible healthcare workers from 34 polyclinics, all agreed to participate and completed the questionnaire. After data validation, 832 participants (79%) were included in the final analysis, while 221 participants (21%) with incomplete or inconsistent data were excluded. Although the sampling was not stratified by occupation, more nurses participated due to the typical 2:1 nurse-to-physician staffing ratio.

A power calculation was performed to determine if 832 sample size of the study was large enough to detect the proportion of providers with adequate knowledge of HPV transmission and disease (correctly answering >70% of the questions). When prevalence of exposure among reference group is 0.3, power becomes greater than 80% when OR is 1.51 or greater. With a fixed sample size of 832, when estimating proportions ranging from 0.1 to 0.5 with 95% confidence level, the margin of error varies between 0.02 and 0.034, corresponding to a range of ±2% to ±3.4%. Therefore, sample size was sufficient to reliably detect the level of knowledge in the study population.

Participants were excluded from the study if they completed the questionnaire in less than 10 minutes, as it was deemed unlikely that they had thoroughly read all the questions. They were also excluded if they provided identical responses to several pairs of logically opposite questions. The pairs considered for this criterion included:

1. “Does the vaccine cause infertility?”
2. “Do you believe the HPV vaccine is safe?”
3. “Do you agree with the statement: ‘HPV vaccination is much more dangerous than HPV itself?’”
4. “Would you vaccinate your own children?”

### Survey tool

Data were collected using an anonymous, structured, self-administered questionnaire in Kazakh and Russian with no personal identifying information developed with KoboToolbox (Kobo Inc., www.kobotoolbox.org).

The survey instrument included socio-demographic questions and questions assessing knowledge and practices related to both routine childhood immunizations and HPV vaccination (Supplementary Table S1).

Before deployment, the questionnaire was pre-tested with a pilot group of healthcare workers. Feedback and observations from this pilot were used to refine the questionnaire. Data from the pilot were not included in the final dataset.

All participants were invited to the polyclinics’ conference rooms for the self-administered online interview A trained survey team of Central Asia FETP residents facilitated group survey sessions, residents provided internet and tablets for data collection to those who needed them, explained the study purpose, obtained written informed consent, and remained available to answer questions or resolve technical issues The polyclinic leadership was asked not to be present at the study venue to reduce response bias.

### Outcomes of interest

Healthcare providers willingness to recommend the HPV vaccine was measured using three questions:

1. Would you have your child vaccinated against HPV?
2. Will you recommend HPV vaccination to children of relatives and friends?
3. Will you recommend HPV vaccination to your patients?

The responses to these questions were classified as:

- Will not recommend: answered “Fully disagree” or “Disagree” to all questions;
- Will recommended: answered “Fully agree” or “Agree” to question three and one or both of questions one or two;
- Neutral: did not fall into either of the above categories

Neutral responses were categorized together with “will not recommend” for the binomial outcome in logistic regression (will recommend vs not) and retained as a separate category in multinomial regression.

Knowledge (K) questions were scored as “1-correct,” “0-incorrect,” or “0-difficult to answer.” Individual respondents’ correct scores on knowledge questions were summarized and then categorized into adequate and inadequate, with scores of 70% or higher defined as adequate (Supplementary Table S1).

### Statistical methods

Data were cleaned and analyzed using R version 4.3.1 (R Foundation for Statistical Computing, Vienna, Austria).

We summarized knowledge, and practice characteristics as proportions. Consistent with standard practice in knowledge testing, neutral responses were treated as incorrect because they signify insufficient or indeterminate knowledge rather than correct comprehension. We assessed the internal consistency of knowledge questions of the questionnaire using Cronbach’s alpha. The internal consistency of composite knowledge scores ranged from 0.50–0.66. Lower internal consistency is common in short factual knowledge tests and reflects content heterogeneity rather than measurement error. The combined scores still provide a meaningful indicator of overall knowledge for use in the regression models.

The chi-square test was used to assess the statistical significance of group differences. Effect sizes were calculated using risk differences (percentage-point differences) and Cohen’s h for differences in proportions. Bivariate logistic regression was performed to calculate odds ratios (ORs) and 95% confidence intervals (CIs) for factors associated with willingness to recommend HPV vaccination and adequate KAP. A p-value < .05 was considered statistically significant. Variables with *p* < .05 in bivariate analysis, along with potential confounders (age, sex, occupation, work experience), were included in multivariable logistic regression models. Model parameters were estimated using maximum likelihood with a logit link. Multicollinearity among predictors was assessed using variance inflation factors (VIF), and GVIF-adjusted VIF values were used for categorical predictors with more than one degree of freedom. Overall model fit was assessed using a likelihood ratio test comparing the full model with the intercept-only model. Model calibration was evaluated using the Hosmer – Lemeshow goodness-of-fit test, and explanatory power was summarized using Nagelkerke’s R^2^. Model discrimination and predictive performance were further evaluated by calculating classification accuracy based on predicted probabilities. Regression coefficients (β) are reported as log-odds with standard errors (SE), Wald test statistics, and p-values. For interpretability, adjusted odds ratios (OR = exp[β]) and 95% confidence intervals were computed using the formula exp(β ± 1.96 × SE). A multinomial logistic regression model was additionally estimated to examine predictors of HPV vaccination recommendation across the three response categories (“not recommend,” “mostly neutral,” and “will recommend”). The same set of demographic and knowledge variables was included as predictors. All analyses were conducted in R using the stats, car, pscl, broom, and gtsummary packages.

## Results

### Participant characteristics

Among the 1057 primary healthcare workers involved in vaccination across the 34 participating polyclinics and present at their workplace during the study period, all completed the survey. We included 832 (79%) healthcare workers in the study, while records for 221 (21%) participants were excluded after data validation. Among 832 study participants, 566 were nurses (68%), 206 (25%) were family physicians, and 60 (7%) were pediatricians (Table 1). The majority (93%) of participants were female, 37% were 27–35 y old, 42% had never had children, 41% had children < 18 y old and 22% lived with someone > 65 y old. Over half (67%) had 0–10 y of working experience, and 22% worked in clinics with catchment populations of >1700 people. Half (47%) of respondents had been screened for cervical cancer, and 4% had a family history of cervical cancer.

**Table 1.** Characteristics of participants in the Human Papillomavirus (HPV) vaccine study among primary care providers, Almaty, Kazakhstan, 2023.

Characteristics	N = 832%)
Occupation	
General doctor	206 (25%)
Pediatrician	60 (7%)
Nurse	566 (68%)
Sex (female)	775 (93%)
Age group	
18–26	237 (29%)
27–35	304 (37%)
36–70	291 (35%)
Marital status	
Married	522 (63%)
Single	253 (30%)
Divorced	57 (7%)
Number of children	
None	347 (42%)
One	120 (14%)
Two	163 (20%)
Three or more	202 (24%)
Has children < 18 y old	342 (41%)
Lives with someone > 65 y old	180 (22%)
Length of professional career (y)	
0–10	557 (67%)
11–20	122 (15%)
>20	153 (18%)
Catchment population that they serve	
<1000	248 (30%)
1000–1700	398 (48%)
>1700	186 (22%)
Had COVID-19	318 (38%)
Has a vaccine contraindication	67 (8%)
Ever screened for cervical cancer	387 (47%)
Ever screened for HPV	173 (21%)
Any family history of cervical cancer	29 (4%)

### Childhood immunization knowledge

In questions about common myths about routine childhood immunization, 39% of participants had adequate knowledge (Table 2). Over half (54%) of participants knew that the pertussis vaccine does not cause sudden death in infants, and 54% knew that there is no causal link between vaccines and autism or multiple sclerosis. Nearly two-thirds (61%) had adequate knowledge about contraindications to routine childhood immunizations. Specifically, 49% knew that uncontrolled seizures or progressive encephalopathy are contraindications, and 66% knew that current antibiotic use is not a contraindication for vaccination.

**Table 2.** Healthcare provider knowledge about childhood immunizations and Human Papillomavirus (HPV), Almaty, Kazakhstan, 2023.

Characteristics	N = 832%)
**Vaccine myths – knowledge score > 70% correct (α = 0.50)**	**320 (39%)**
Knows that the influenza vaccine does not cause influenza	382 (46%)
Knows that simultaneous administration of several vaccines does not lead to an immune system overload	396 (48%)
Knows that the pertussis vaccine does not cause sudden infant death syndrome	452 (54%)
Knows that there is no causal link between getting vaccines and autism or multiple sclerosis	453 (54%)
**Vaccine contraindications – knowledge score > 70% correct (α = 0.55)**	**505 (61%)**
Knows that uncontrolled seizures or progressive encephalopathy are a contraindication	404 (49%)
Knows that current antibiotic use is not a contraindication for vaccination	547 (66%)
Knows that moderate or severe acute illness with or without fever is a precaution to vaccine administration	627 (75%)
Knows that a severe allergic reaction to a previous dose is a contraindication	655 (79%)
Knows that fever alone is not necessarily a contraindication	682 (82%)
**HPV transmission and disease – knowledge score > 70% correct (α = 0.66)**	**272 (33%)**
Knows that there is currently no cure for HPV	181 (22%)
Knows that the HPV vaccine can prevent cervical cancer	480 (58%)
Knows that HPV infection causes meningitis	540 (65%)
Knows that HPV infection can cause cervical cancer	575 (69%)
Knows that HPV is not an airborne infection	588 (71%)
Knows that HPV is transmitted by direct contact	615 (74%)
Knows that HPV infection can cause cancer of the genital organs	631 (76%)

α = Cronbach’s alpha.

### HPV disease and vaccine knowledge and practice

In questions about HPV transmission and disease (Table 2), 33% of participants had adequate knowledge. Only 22% of participants knew that there is currently no cure for HPV, 58% knew that the HPV vaccine can prevent cervical cancer, and 71% knew that HPV is not an airborne disease.

**Figure 1.**
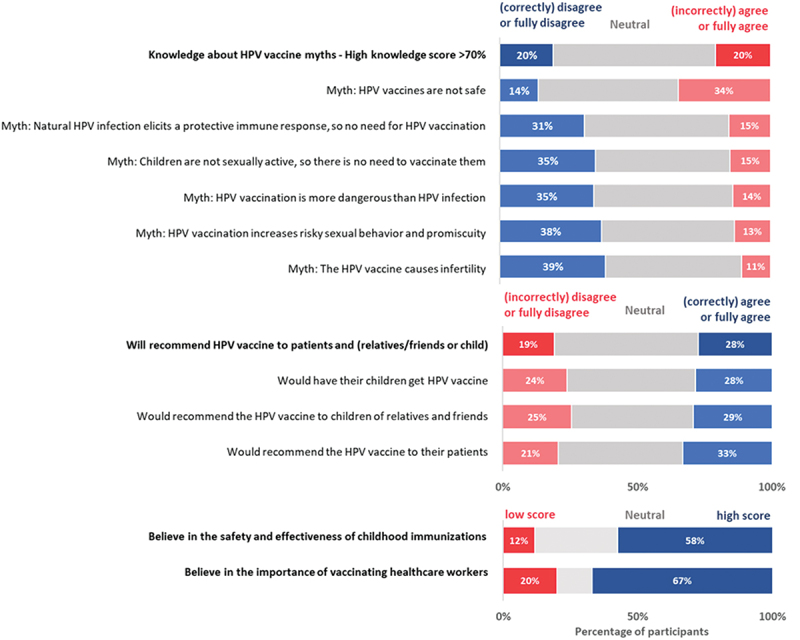
HPV vaccine knowledge, vaccine perceptions, and recommendation practices among primary care providers, Almaty, Kazakhstan, 2023.

In questions related to HPV vaccine myths, 20% of participants were able to correctly dispel common myths (with scores of >70%) and 60% selected neutral responses to all HPV vaccine myths (Figure 1). Specifically, 35% could correctly dispel the myth that HPV vaccination is more dangerous than HPV disease, and 39% could correctly dispel the myth that HPV vaccination causes infertility.

One-third (33%) of providers would recommend the HPV vaccine to their patients, 29% to family and friends, and 28% to their children. Overall, 28% would recommend the vaccine to their patients and to their own children or children of their relatives and friends. Meanwhile, 53% of providers were neutral about recommending HPV vaccination for their patients, relatives, friends or own children.

Over half (58%) of participants agreed or strongly agreed in the safety and effectiveness of childhood vaccines, and 67% agreed or strongly agreed that routine immunization for healthcare providers was important for protecting themselves, their families, and their patients.

### Occupational differences

Adequate knowledge about childhood vaccines was significantly higher among physicians than nurses (50% vs 33%, respectively, *p* < .01) (Table 3). The proportion of physicians with adequate knowledge of HPV transmission and disease was nearly twice that of nurses (47% vs 26%, respectively, *p* < .01). Compared to nurses, a higher proportion of physicians had adequate knowledge about HPV vaccine myths (27% compared to 17% among physicians and nurses, respectively, *p* < .01).

**Table 3.** Healthcare provider occupation and knowledge about childhood immunizations and HPV, Almaty, Kazakhstan, 2023.

Characteristics	Physician	Nurse		Risk Diff**	Cohen’s h
	(N = 266)	(N = 566)	P*		
Childhood vaccine myths – Knowledge score > 70% correct	133 (50%)	187 (33%)	<.01	17	0.35
Childhood vaccine contraindications – Knowledge score > 70% correct	181 (68%)	324 (57%)	<.01	10.8	0.22
HPV transmission and disease – Knowledge score > 70% correct	125 (47%)	147 (26%)	<.01	21	0.44
Will recommend HPV vaccine to patients and (relatives/friends or child)					
Won’t recommend	36 (14%)	123 (22%)	<.01	−8.2	−0.22
Mostly neutrals	136 (51%)	308 (54%)		−3.3	−0.07
Will recommend	94 (35%)	135 (24%)		11.5	0.25
Knowledge about HPV vaccine myths					
Low score (<70% correct)	39 (15%)	125 (22%)	<.01	−7.4	−0.19
Mostly neutral responses	154 (58%)	345 (61%)		−3.1	−0.06
High score (>70% correct)	73 (27%)	96 (17%)		10.5	0.2

HPV- Human Papillomavirus.

*Chi-square test *p*-value.

** Risk Diff- risk difference.

### Factors associated with the willingness to recommend the HPV vaccine

In multivariable analysis, we found that physicians had 52% increased odds of recommending the HPV vaccine compared to nurses (OR: 1.52, 95% CI: 1.06–2.18) (Table 4). Regardless of their specialty, providers with adequate knowledge about childhood vaccine myths had 47% greater odds of recommending the vaccine than providers who could not (OR: 1.47, 95% CI: 1.03–2.07). Odds of recommending the vaccine were also greater for providers who had adequate knowledge of HPV disease compared to providers with inadequate knowledge (OR: 1.63, 95% CI: 1.14–2.32), who believed in the safety and effectiveness of routine childhood vaccines compared to providers who did not (OR: 1.65, 95% CI: 1.12–2.47), and who believed in the importance of vaccinating healthcare workers than providers who did not (OR: 3.07, 95% CI: 2.11–4.54). Model calibration was assessed using the Hosmer – Lemeshow test, which indicated good fit (χ^2^ = 2.84, df = 8, *p* = .94). A likelihood ratio test indicated that the full logistic regression model provided a significantly better fit than the null model (likelihood ratio χ^2^ = 107.48, df = 8, *p* < .001), suggesting that the included predictors collectively explain variation in HPV vaccination practices.

**Table 4.** Factors associated with the HPV vaccination recommendation behavior among healthcare providers, Almaty, Kazakhstan, 2023.

Characteristics	Providers N	Will recommend the vaccine n (%)	aOR	95% CI	*p*-value
Age group (in y)					
18–26	237	58 (24%)	Ref		
27–35	304	80 (26%)	1.06	0.67–1.68	.800
36–70	291	91 (31%)	1.19	0.77–1.85	.400
Occupation					
Nurse	566	135 (24%)	Ref		
Physician	266	94 (35%)	1.52	1.06–2.18	.024
Have children < 18 y old					
Yes	342	91 (27%)	Ref		
No	490	138 (28%)	1.23	0.86–1.76	.300
Can correctly dispel common myths about childhood vaccines					
No	512	106 (21%)	Ref		
Yes	320	123 (38%)	1.47	1.03–2.07	.031
Have adequate knowledge about HPV disease					
No	560	119 (21%)	Ref		
Yes	272	110 (40%)	1.63	1.14–2.32	.007
Believe in the safety and effectiveness of other routine childhood vaccines					
No	275	44 (16%)	Ref		
Yes	557	185 (33%)	1.65	1.12–2.47	.013
Believe in vaccinating healthcare providers					
No	353	46 (13%)	Ref		
Yes	479	183 (38%)	3.07	2.11–4.54	<.001

HPV- Human Papillomavirus.

aOR = adjusted Odds Ratio, CI = Confidence Interval

Multivariable logistic regression model. Adjusted odds ratios (OR = exp(β)) and 95% confidence intervals are reported; 95% CIs are calculated as exp(β ± 1.96 × SE). *P*-values are derived from Wald tests for each coefficient. Overall model fit was significant (likelihood ratio χ^28^ = 107.48, *p* < .001), and model calibration was adequate (Hosmer – Lemeshow goodness-of-fit test: χ^28^ = 2.84, *p* = .94). All predictors showed minimal multicollinearity, with GVIF-adjusted VIF values near 1.0, indicating that multicollinearity was not a concern in this model. Nagelkerke’s R^2^ was 0.175, indicating that the model explained approximately 17.5% of the variance in HPV vaccination practices.

The results of the multinomial logistic regression were highly consistent with those of the binary logistic model (Supplementary Table S2). In both analyses, being a physician (vs. nurse), having stronger vaccine- and HPV-related knowledge, and positive vaccine perceptions were associated with higher odds of recommending the HPV vaccine. Belief in vaccinating healthcare providers remained the strongest predictor across models. The multinomial model distinguished between “mostly neutral” and “will recommend,” but the direction and significance of effects were similar across categories, with slightly larger effect sizes in the “will recommend” group. Overall, the two models produced parallel findings, indicating robust and stable associations regardless of whether recommendation behavior was treated as binary or multinomial.

## Discussion

Our study aimed to assess the healthcare workers’ knowledge of vaccines and intent to recommend the HPV vaccine in Almaty, Kazakhstan, shortly before the reintroduction of HPV vaccination into the national immunization schedule. We identified significant gaps in healthcare providers’ knowledge, and practices related to childhood immunizations and the HPV vaccine. Notably, only two in five providers were able to dispel common myths about childhood immunizations, and over one-third could not adequately identify vaccine contraindications. For HPV vaccines, only one in five providers could refute common myths, and about one-quarter expressed a willingness to recommend the HPV vaccine.

The proportion of providers willing to recommend the HPV vaccine in our study is comparatively lower than in other published studies, falling below the pooled prevalence of 60% reported in a meta-analysis of 35 studies.^27^ However, our findings align with the pooled prevalence of 19% observed in studies conducted in the Eastern Mediterranean region. Additionally, our results are lower than those from a 2023 study by Kasymbekova et al. in Kazakhstan, which found that 73% of 1189 healthcare professionals expressed a positive intention to recommend the HPV vaccine.^28^ This discrepancy may be due to the differences in study design: our research focused specifically on primary care providers responsible for routine childhood and adolescent HPV vaccinations in all public and private outpatient clinics in Almaty. In contrast, Kasymbekova et al. included providers from all specialties, regardless of their role in vaccination, and used snowball sampling through messaging apps, social media, and official mailings.^29^

Vaccine hesitancy is a major barrier to HPV vaccination programs worldwide.^18,19^ Hesitancy has five interrelated components: trust, convenience, complacency, communication, and context. Trust refers to confidence in vaccines, providers, and institutions; convenience to availability, accessibility, and affordability; complacency to low perceived risk of diseases; communication to the quality and reach of information; and context to broader social, cultural, and political influence. Our findings suggest that all five components are relevant in Kazakhstan.

The large neutral group of response in our sample is particularly noteworthy and suggests ambivalence rather than outright opposition toward HPV vaccination. Over half of providers in this study selected neutral responses to all HPV vaccine myths; while only a small minority of providers explicitly rejected the statement that HPV vaccines are not safe, and about one-fifth of providers supported several vaccine-related myths. This large neutral group in our study may represent a “hesitancy of uncertainty,” in which providers neither actively oppose nor confidently support HPV vaccination. These findings underscore the importance of combining HPV vaccine introduction with educational outreach to healthcare providers, rather than relying on passive dissemination of information alone. The high proportion of neutral responses indicates that even long-established vaccines on the international market may not be adequately understood without active education efforts targeting healthcare providers.

The low level of knowledge about HPV disease and vaccine among healthcare providers in our study is consistent with findings from the same Kazakhstan study, where only 17% of providers had adequate knowledge. Other studies conducted in Kazakhstan and neighboring countries have similarly reported low knowledge levels about childhood immunizations among healthcare workers.^30–32^ Low knowledge levels and widespread myths point to challenges in trust and communication, particularly where misinformation about vaccine safety, infertility, and sexual behavior circulates more effectively than evidence-based messages.^15,18–21^

As mentioned in one of these studies, the low knowledge level among providers might be attributed to the fact that medical education in Kazakhstan continues to rely heavily on Russian-language Soviet textbooks published prior to 1991 without much if any, training on vaccine safety, effectiveness, and communicating strategies with vaccine hesitant parents to, and on searching and using peer-reviewed literature on vaccine safety and effectiveness.”^33^

The literature consistently demonstrates that healthcare providers who possess knowledge about HPV and vaccines are more likely to recommend vaccination to their patients. Furthermore, educational interventions have proven effective in increasing providers’ willingness to recommend HPV vaccines.^34,35^ Therefore, increasing vaccine knowledge among providers in Kazakhstan may lead to a higher number of practitioners recommending the vaccine to their patients and communities. Modernizing immunization training materials in medical schools in Kazakhstan and providing continuing education for healthcare providers on immunization and communication with vaccine-hesitant populations may strengthen provider capacity.^30,33,34^ Embedding HPV vaccination content into undergraduate medical and nursing curricula and residency programs could contribute to a stronger foundation for future providers. Interpersonal communication training – addressing parental concerns, countering misinformation, and delivering strong, presumptive vaccine recommendations – may improve translation of knowledge into practice. Leveraging local champions, peer-to-peer education, and context-specific materials (e.g., in Kazakh and Russian languages, tailored to prevalent myths in Kazakhstan) may further increase impact.

The knowledge gap by occupation found in our study was anticipated. Previous studies in Kazakhstan and the surrounding region have similarly indicated that nurses generally possess lower knowledge about vaccines and HPV compared to physicians^30,36,37^ In Kazakhstan, nurses comprise the majority of primary healthcare workers and play a key role in the vaccination process.^38,39^ Primary healthcare nurses are responsible for reminding caregivers when their children are due for vaccinations, serving as gatekeepers to fostering vaccine confidence among patients. A nurse’s positive attitude toward vaccination and adherence to best health practices can increase parents’ willingness to vaccinate their children.^40–42^ Consequently, vaccination coverage could improve if nurses consistently and correctly inform parents about vaccines and recommend vaccinations.

Although we did not assess the correlation between individual providers’ knowledge scores and vaccine coverage levels, our study showed that intent to recommend the HPV vaccine had a positive association between beliefs in the safety and effectiveness of routine childhood vaccines and the importance of vaccination for healthcare workers. These findings are similar to previous study in Kazakhstan, which showed that low confidence in COVID-19 vaccines was positively associated with poor overall knowledge of routine childhood vaccines and negative general attitudes toward vaccination. This association was also evident in misconceptions about the safety and effectiveness of both routine and COVID-19 vaccines.^30^ However, knowledge of childhood vaccines did not necessarily translate into confident support for newer vaccines, such as the HPV vaccine. This discrepancy may explain why gaps in HPV vaccination coverage persist in settings with relatively high coverage of other childhood vaccines.

Our study is subject to several limitations. First, because it was conducted solely in Almaty, the findings may not be representative of all primary care workers in Kazakhstan, particularly those in rural or remote areas. Second, administering the survey directly at participant workplaces may introduce social desirability bias, potentially leading to overestimates of positive believes and willingness to recommend the HPV vaccine. To mitigate this effect, we ensured that the surveys were self-administered and anonymous. Facilities leaderships were asked not to be present during survey. Third, the cross-sectional design limits causal inference; we cannot conclude that increasing knowledge would result in higher recommendation rates, although this is supported by previous interventional studies.^33,34^ Finally, while we conducted sensitivity analyses to explore the impact of respondents with uniformly neutral believes, residual uncertainty remains regarding how best to interpret neutrality in the context of vaccine recommendation.

In conclusion, our study revealed alarmingly low levels of knowledge, and practices related to HPV and other childhood immunizations among healthcare providers, particularly nurses, few months before the introduction of HPV vaccination in Kazakhstan. This low level of knowledge was associated with decreased willingness to recommend HPV vaccines. Our findings, along with other studies, has highlighted the need for supplemental training for healthcare providers on HPV vaccination in Kazakhstan to support the implementation of a national rollout strategy.^30^ The Ministry of Health of Kazakhstan conducted extensive capacity-building activities in 2023–2024, including over 15 national and regional training events for healthcare providers – reaching more than 2000 pediatricians, family physicians, epidemiologists, gynecologists, nurses, and communication specialists. As of April 2025, HPV vaccination coverage among targeted girls aged 11 to 13 had reached 40%, still short of the WHO target of 90%. Increasing HPV vaccination coverage will require ongoing efforts to increase public awareness, improve provider training, reform medical and nursing education to integrate vaccination practices better, and strengthen community engagement.

## Supplementary Material

Supplemental Material

## Data Availability

The datasets generated for this study are available on request from the corresponding author.

## References

[1] World Health Organization. The cancer we can eliminate – WHO/Europe urges member states to consign cervical cancer to history [Electronic resource]. 2022 Sep 13. https://www.who.int/europe/news/item/13-09-2022-the-cancer-we-can-eliminate—who-europe-urges-member-states-to-consign-cervical-cancer-to-history.

[2] Global cancer burden growing, amidst mounting need for services [Internet]. [accessed 2025 Apr 24]. https://www.who.int/news/item/01-02-2024-global-cancer-burden-growing–amidst-mounting-need-for-services. PMC11115397 38438207

[3] World Health Organization. Global strategy to accelerate the elimination of cervical cancer as a public health problem. World Health Organization. Licence: CC BY-NC-SA 3.0 IGO; 2020. https://www.who.int/publications/b/55745.

[4] Ministry of Health of the Republic of Kazakhstan. Cervical cancer can be prevented through HPV vaccination – Chief Oncologist of the Ministry of Health of the Republic of Kazakhstan. Electronic resource. 2025 Apr 8 [accessed 2025 Apr 8]. https://www.gov.kz/memleket/entities/dsm/press/article/details/111112?lang=ru.

[5] Schiller JT, Castellsagué X, Garland SM. A review of clinical trials of human papillomavirus prophylactic vaccines. Vaccine [Internet]. 2012 [accessed 2025 Apr 8]. 30 Suppl 5(5). https://pubmed.ncbi.nlm.nih.gov/23199956/. 10.1016/j.vaccine.2012.04.108 PMC4636904 23199956

[6] Munoz N, Kjaer SK, Sigurdsson K, Iversen OE, Hernandez-Avila M, Wheeler CM, Perez G, Brown DR, Koutsky LA, et al. Impact of human papillomavirus (HPV)-6/11/16/18 vaccine on all HPV-associated genital diseases in young women. JNCI: J Natl Cancer Inst [Internet]. 2010 Mar 3 [accessed 2024 June 11]. 102(5):325–14. doi: 10.1093/jnci/djp534. 20139221

[7] Monsonego J, Cortes J, Greppe C, Hampl M, Joura E, Singer A. Benefits of vaccinating young adult women with a prophylactic quadrivalent human papillomavirus (types 6, 11, 16 and 18) vaccine. Vaccine [Internet]. 2010 Nov 29 [accessed 2024 June 11]. 28(51):8065–8072. https://pubmed.ncbi.nlm.nih.gov/20971114/. 20971114 10.1016/j.vaccine.2010.10.017

[8] Falcaro M, Castañon A, Ndlela B, Checchi M, Soldan K, Lopez-Bernal J, Elliss-Brookes L, Sasieni P. The effects of the national HPV vaccination programme in England, UK, on cervical cancer and grade 3 cervical intraepithelial neoplasia incidence: a register-based observational study. Lancet [Internet]. 2021 Dec 4 [accessed 2025 Apr 21]. 398(10316):2084–2092. https://pubmed.ncbi.nlm.nih.gov/34741816/. 34741816 10.1016/S0140-6736(21)02178-4

[9] Dorali P, Damgacioglu H, Clarke MA, Wentzensen N, Orr BC, Sonawane K, Deshmukh AA. Cervical cancer mortality among US women younger than 25 years, 1992-2021. JAMA [Internet]. 2025 Nov 27 [accessed 2025 Apr 21]. 333(2):165. https://pubmed.ncbi.nlm.nih.gov/39602175/. 39602175 10.1001/jama.2024.22169 PMC11733696

[10] Gargano JW, Stefanos R, Dahl RM, Castilho JL, Bostick EA, Niccolai LM, Park IU, Blankenship S, Brackney MM, Chan K, et al. Trends in cervical precancers identified through population-based surveillance - human papillomavirus vaccine impact monitoring project, five sites, United States, 2008-2022. MMWR Morb Mortal Wkly Rep. 2025;74(6):96–101. doi: 10.15585/mmwr.mm7406a4. 40014651 PMC11867585

[11] Viveros-Carreño D, Fernandes A, Pareja R. Updates on cervical cancer prevention. Int J Gynecological Cancer. 2023 Mar. 33(3):394–402. doi: 10.1136/ijgc-2022-003703. 36878567

[12] Rahangdale L, Mungo C, O’Connor S, Chibwesha CJ, Brewer NT. Human papillomavirus vaccination and cervical cancer risk. BMJ. 2022 Dec 15. 379:e070115. doi: 10.1136/bmj-2022-070115. 36521855

[13] Ministry of Health of the Republic of Kazakhstan. On the approval of the sanitary rules “Sanitary and epidemiological requirements for the organization and implementation of preventive vaccinations for the population”. 2023. https://adilet.zan.kz/rus/docs/V2300033463.

[14] Pharmnews.kz. Results of the HPV vaccination effectiveness study in Kazakhstan [Electronic resource]. 2025. https://pharmnewskz.com/ru/news/rezultaty-issledovaniya-effektivnosti-vakcinacii-protiv-vpch-v-kazahstane_22871.

[15] Vlast.kz. How the HPV vaccine “failed” in Kazakhstan [Electronic resource]. 2025. https://vlast.kz/vaccination/46260-kak-v-kazahstane-provalilas-vakcina-ot-vpc.html.

[16] World Health Organization. Global partners cheer progress towards eliminating cervical cancer and underline challenges [Electronic resource]. 2023 Nov 17. https://www.who.int/news/item/17-11-2023-global-partners-cheer-progress-towards-eliminating-cervical-cancer-and-underline-challenges.

[17] World Health Organization. Accelerating the elimination of cervical cancer as a public health problem: towards achieving 90–70–90 targets by 2030 [Electronic resource]. 2022. https://iris.who.int/handle/10665/361138.

[18] Holman DM, Benard V, Roland KB, Watson M, Liddon N, Stokley S. Barriers to human papillomavirus vaccination among US adolescents: a systematic review of the literature. JAMA Pediatr [Internet]. 2014 Jan [accessed 2025 Apr 9]. 168(1):76–82. https://pubmed.ncbi.nlm.nih.gov/24276343/. 24276343 10.1001/jamapediatrics.2013.2752 PMC4538997

[19] Cadeddu C, Castagna C, Sapienza M, Lanza TE, Messina R, Chiavarini M, Ricciardi W, De Waure C. Understanding the determinants of vaccine hesitancy and vaccine confidence among adolescents: a systematic review. Hum Vaccin Immunother [Internet]. 2021 [accessed 2025 Apr 9]. 17(11):4470–4486. https://pubmed.ncbi.nlm.nih.gov/34473589/. 34473589 10.1080/21645515.2021.1961466 PMC8828162

[20] Costantino C, Tabacchi G, Sannasardo CE, Scarpitta F, Vella C, Vitale F, Casuccio A, Restivo V, et al. Systematic review and meta-analysis of determinants associated with HPV vaccination uptake in Europe. Eur J Public Health [Internet]. 2020 Sep 1 [accessed 2025 Apr 9]. 30(Supplement_5). https://www.researchgate.net/publication/347121356_Systematic_review_and_meta-analysis_of_determinants_associated_with_HPV_vaccination_uptake_in_Europe.

[21] Sannasardo CE, Costantino C, Tabacchi G, Sannasardo CE, Scarpitta F, Vella C, Vitale F, Casuccio A, Restivo V, et al. Systematic review and meta-analysis of determinants associated with HPV vaccination uptake in Europe. Eur J Public Health [Internet]. 2020 Sep 1 [accessed 2025 Apr 8]. 30(Supplement_5). 10.1093/eurpub/ckaa166.1438.

[22] Alaba Awolude O, Oyerinde SO. Assessment of infrastructural and human resource for health status and readiness for HPV vaccination in rural communities in Nigeria. J Cancer Sci Clin Ther. 2019;3(3). doi: 10.26502/jcsct.5079021.

[23] Akova İ, Koşaroğlu NE, Kılıç E. Knowledge, attitudes, and behaviours of primary health care workers regarding HPV infection and prevention: an example from Türkiye. Turk J Fam Med Primary Care [Internet]. 2023 Sep 20 [accessed 2025 Apr 9]. 17(3):407–415. https://dergipark.org.tr/en/pub/tjfmpc/issue/79870/1310981.

[24] Mao Y, Zhao Y, Zhang L, Li J, Abdullah AS, Zheng P, Wang F. Frequency of health care provider recommendations for HPV vaccination: a survey in three large cities in China. Front Public Health [Internet]. 2023 Jul 11 [accessed 2025 Apr 9]. 11:1203610. https://www.wjx.cn/app/survey.aspx. 37497028 10.3389/fpubh.2023.1203610 PMC10366465

[25] Babi A, Issa T, Issanov A, Akhanova S, Udalova N, Koktova S, Balykov A, Sattarkyzy Z, Imankulova B, Kamzayeva N, et al. Knowledge and attitudes of mothers toward HPV vaccination: a cross-sectional study in Kazakhstan. Womens Health (Lond) [Internet]. 2023 Jan 1 [accessed 2025 Apr 8]. 19. https://pubmed.ncbi.nlm.nih.gov/37184051/. 10.1177/17455057231172355 PMC10192804 37184051

[26] Resolution of the Chief State Sanitary Doctor of the Republic of Kazakhstan dated September 17, 2024, No. 11. On the organization and implementation of vaccination against human papillomavirus in the Republic of Kazakhstan. n.d [accessed 2025 Apr 9]. https://online.zakon.kz/Document/?doc_id=33404547&pos=3;-71#pos=3;-71.

[27] Bakare D, Gobbo E, Akinsola KO, Bakare AA, Salako J, Hanson C, Herzig van Wees S, Falade A, King C. Healthcare worker practices for HPV vaccine recommendation: a systematic review and meta-analysis. Hum Vaccin Immunother [Internet]. 2024 Dec 31 [accessed 2025 Apr 8]. 20(1):2402122. https://pubmed.ncbi.nlm.nih.gov/39400296/. 39400296 10.1080/21645515.2024.2402122 PMC11486212

[28] Kassymbekova F, Rommel A, Kaidarova D, Auyezova A, Nukusheva S, Dunenova G, Bolatbekova R, Zhetpisbayeva I, Abdushukurova G, Glushkova N. Developing HPV vaccination communication strategies: assessing knowledge, attitudes, and barriers among healthcare professionals in Kazakhstan. Vaccines (Basel) [Internet]. 2024 Nov 1 [accessed 2025 Apr 8]. 12(11):1225. https://www.mdpi.com/2076-393X/12/11/1225/htm. 39591128 10.3390/vaccines12111225 PMC11598784

[29] Kassymbekova F, Zhetpisbayeva I, Tcoy E, Dyussenov R, Davletov K, Rommel A, Glushkova N. Exploring HPV vaccine knowledge, attitudes, barriers and information sources among parents, health professionals and teachers in Kazakhstan: a mixed-methods study protocol. BMJ Open [Internet]. 2023 Sep 22 [accessed 2025 Apr 21]. 13(9):e074097. https://pubmed.ncbi.nlm.nih.gov/37739465/. 10.1136/bmjopen-2023-074097 PMC10533667 37739465

[30] Nabirova D, Horth R, Kassabekova L, Henderson A, Yesmagambetova A, Alaverdyan S, Nuorti JP, Smagul M. Factors associated with COVID-19 vaccine confidence among primary care providers in Kazakhstan, March–April 2021. Front Public Health. 2023 Sep 7. 11:1245750. doi: 10.3389/fpubh.2023.1245750. 37744481 PMC10517263

[31] Akmatova R, Dzhangaziev B, Ebama MS, Otorbaeva D. Knowledge, attitudes, and practices (KAP) towards seasonal influenza and influenza vaccine among pregnant women in Kyrgyzstan: a cross-sectional study. Vaccine. 2024 Oct 24. Suppl 42:125510. doi: 10.1016/j.vaccine.2023.12.020. Epub 2023 Dec 9. PMID: 38072755. 38072755

[32] Naranzul N, Burmaajav B, Burmaajav B, Enkhjargal A, Tumurbat B, Amgalan B, Suvd B, Khurelbaatar N, Baatarkhuu O, Naranzul N, et al. Knowledge, attitudes, and practices (KAP) regarding hepatitis B vaccination among healthcare workers in Mongolia. Occup Dis Environ Med. 2023;11(1):30–48. doi: 10.4236/ODEM.2023.111002.

[33] Yessirkepov M, Nurmashev B, Anartayeva M. A Scopus-based analysis of publication activity in Kazakhstan from 2010 to 2015: positive trends, concerns, and possible solutions. J Korean Med Sci. 2015;30(12):1915. doi: 10.3346/JKMS.2015.30.12.1915. 26713071 PMC4689840

[34] Leung SOA, Akinwunmi B, Elias KM, Feldman S. Educating healthcare providers to increase human papillomavirus (HPV) vaccination rates: a qualitative systematic review. Vaccine: X. 2019 3. 3:100037. doi: 10.1016/J.JVACX.2019.100037. 31463471 PMC6708991

[35] Chen H, Zhang X, Wang W, Zhang R, Du M, Shan L, Li Y, Wang X, Liu Y, Zhang W, et al. Effect of an educational intervention on human papillomavirus (HPV) knowledge and attitudes towards HPV vaccines among healthcare workers (HCWs) in western China. Hum Vaccin Immunother. 2021;17(2):443–450. doi: 10.1080/21645515.2020.1780093. 32692948 PMC7899665

[36] Nishioka H, Onishi T, Kitano T, Takeyama M, Imakita N, Kasahara K, Kawaguchi R, Masaki JA, Nogami K. A survey of healthcare workers’ recommendations about human papillomavirus vaccination. Clin Exp Vaccine Res. 2022;11(2):149. doi: 10.7774/CEVR.2022.11.2.149. 35799873 PMC9200650

[37] Aggarwal I, Mehta D, Yadav P, Rakheja S, Goel H, Vinod A, Knowledge DD. Attitude and practice on human papillomavirus vaccination among healthcare providers at a tertiary care centre in North Delhi. Asian Pac J Cancer Prev. 2025;26(2):671–676. doi: 10.31557/APJCP.2025.26.2.671. 40022716 PMC12118011

[38] Llop-Gironés A, Azhymambetova GK, Asanova AK, Salomuddin Y, Boynazarova MH, Raupov FO, Zholzhanova NU, Ruzdenova NB, Tojiboyeva GS, Salikhodjayeva RK, et al. Building health systems resilience in Central Asia through nursing and midwifery: evidence to inform policy action. Hum Resour Health. 2024;22(1):1–10. doi: 10.1186/s12960-024-00964-3. 39696577 PMC11654295

[39] World Health Organization. Roadmap for health and well-being in Central Asia (2022–2025). Electronic resource. 2022. https://www.who.int/europe/publications/i/item/WHO-EURO-2022-5905-45670-65601.

[40] Smith PJ, Kennedy AM, Wooten K, Gust DA, Pickering LK. Association between health care providers’ influence on parents who have concerns about vaccine safety and vaccination coverage. Pediatrics. 2006;118(5):e1287–e1292. doi: 10.1542/PEDS.2006-0923. 17079529

[41] Hoekstra S, Margolis L. The importance of the nursing role in parental vaccine decision making. Clin Pediatrics. 2016;55(5):401–403. doi: 10.1177/0009922815627348. 26810621

[42] Santa Maria D, Markham C, Misra SM, Coleman DC, Lyons M, Desormeaux C, Cron S, Guilamo-Ramos V. Effects of a randomized controlled trial of a brief, student-nurse led, parent-based sexual health intervention on parental protective factors and HPV vaccination uptake. BMC Public Health. 2021 21. 21(1). doi: 10.1186/S12889-021-10534-0. PMC7992324 33761920

