# Supplementary material for: Human Papillomavirus vaccine knowledge and recommendation practice among primary care providers, Almaty, Kazakhstan – 2023
Source: Hum Vaccin Immunother. 2026 Feb 2;22(1):2610622. doi: 10.1080/21645515.2025.2610622 (PMC12867355; doi:10.1080/21645515.2025.2610622)
Supplement: Supplemental Material [file KHVI_A_2610622_SM2575.pdf]

Supplementary Table 1. Questions used for evaluating HPV knowledge, vaccine perceptions, and recommendation practices

Domain	Questions
**HPV transmission and disease (>70% correct) (7 questions)**	HPV infection leads to cervical cancer. Correct response: YES
	HPV infection causes cancer of the genital organs. Correct response: YES
	HPV infection causes meningitis. Correct response: NO
	HPV is an airborne infection. Correct response: NO
	HPV is transmitted by direct contact. Correct response: YES
	There is currently a cure for HPV. Correct response: NO
	HPV vaccine can prevent cervical cancer. Correct response: YES
**Vaccine myths (>70% correct) (4 questions)**	Can simultaneous administration of several vaccines lead to an overload of the immune system? Correct response = NO
	Current scientific evidence supports a link between getting vaccines and diseases like autism and multiple sclerosis? Correct response = NO
	Can pertussis vaccine cause sudden infant death syndrome? Correct response = NO
	Can the flu vaccine cause the flu? Correct response = NO
**HPV vaccine myths (>70%) (6 questions)**	The HPV vaccine causes infertility. Correct response: DISAGREE
	Natural HPV infection already elicits a protective antibody response, so there is no need for HPV vaccination. Correct response: DISAGREE
	Children are not sexually active, so there is no need to vaccinate them against something that does not concern them at this age. Correct response: DISAGREE
	HPV vaccination increases risky sexual behavior and promiscuity. Correct response: DISAGREE
	Do you agree with the statement: "HPV vaccines are more dangerous than HPV infection". Correct response: DISAGREE
	Do you believe the HPV vaccine is safe? Correct response: AGREE
**Safety and effectiveness of childhood immunizations (70%) (16 questions)**	Tuberculosis vaccines are effective.
	HBV vaccines are effective.
	Vaccines against diphtheria, tetanus are effective.
	Vaccines against measles, rubella and mumps are effective.
	Pertussis, diphtheria and tetanus vaccines are effective.
	Polio vaccine are effective.
	Haemophilus influenzae type b vaccines are effective.
	Vaccines against pneumococcal infection are effective.
	Tuberculosis vaccine are safe.
	HBV vaccines are safe.
	Vaccines against diphtheria, tetanus are safe.
	Vaccines against measles, rubella and mumps are safe.
	Pertussis, diphtheria and tetanus vaccine (DTP) are safe.
	Polio vaccine are safe.
	Haemophilus influenzae type b vaccines are safe.
	Vaccines against pneumococcal infection are safe.
**Medical workers and vaccination (70%) (4 questions)**	Do you agree with the statement: I'd rather get sick than get any vaccine
	HCW should get vaccinated to protect their families
	HCW should get vaccinated to protect themselves
	HCW should get vaccinated to protect patients
**Recommending HPV vaccine to patients and family/friends OR child (3 questions)**	Would have their children get HPV vaccine
	Would recommend the HPV vaccine to children of relatives and friends
	Would recommend the HPV vaccine to their patients

Supplementary Table 2. Multinomial logistic regression of factors associated with HPV vaccination recommendation behavior

	Mostly neutrals (vs not recommend)			Will recommend (vs not recommend)		
**Characteristics**	**aOR**	**95% CI**	**p**	**aOR**	**95% CI**	**p**
Age group (in years)						
18-26	Ref			Ref		
27-35	0.97	0.58, 1.61	>0.9	1.04	0.57, 1.89	>0.9
36-70	0.85	0.52, 1.39	0.5	1.06	0.59, 1.88	0.8
Occupation						
Nurse	Ref			Ref		
Physician	1.47	0.94, 2.30	0.09	2.04	1.23, 3.36	0.005
Have children <18 years old						
Yes	Ref			Ref		
No	1.5	1.01, 2.24	0.05	1.67	1.04, 2.66	0.032
Can correctly dispel common myths about childhood vaccines						
No	Ref			Ref		
Yes	1.3	0.85, 1.98	0.2	1.78	1.11, 2.87	0.017
Have adequate knowledge about HPV disease						
No	Ref			Ref		
Yes	1.18	0.76, 1.86	0.5	1.86	1.14, 3.04	0.014
Believe in the safety and effectiveness of other routine childhood vaccines						
No	Ref			Ref		
Yes	1.51	1.02, 2.22	0.04	2.23	1.37, 3.62	0.001
Believe in vaccinating healthcare providers						
No	Ref			Ref		
Yes	0.79	0.54, 1.17	0.2	2.58	1.59, 4.19	<0.001

Note: Results are from a multinomial logistic regression model examining predictors of HPV vaccination recommendation categories, with "will not recommend" as the reference outcome. Adjusted odds ratios (aOR = exp[β]) and 95% confidence intervals were calculated as exp(β ± 1.96 × SE), and p-values were obtained from Wald tests. Overall model fit was supported by a significant likelihood ratio test comparing the full model to the intercept-only model ($\chi^2(16)$ = 124.25, $p < .001$). Pseudo-R^2 statistics indicated modest explanatory power (McFadden = 0.07; Cox–Snell = 0.14; Nagelkerke = 0.16). Multicollinearity diagnostics using GVIF-adjusted VIF values showed no evidence of problematic multicollinearity among predictors (all GVIF^(1/(2×df)) < 2.3). Although mild multicollinearity was observed between vaccine efficacy/safety attitudes and other predictors, the magnitude was not sufficient to threaten model validity or inflate standard errors.
